# Quality of Care for Patients with Type 2 Diabetes Mellitus in Dubai: A HEDIS-Like Assessment

**DOI:** 10.1155/2015/413276

**Published:** 2015-05-24

**Authors:** Shelagh M. Szabo, Katherine M. Osenenko, Lara Qatami, Bonnie M. Korenblat Donato, Ellen E. Korol, Abdulrazzaq A. Al Madani, Fatheya F. Al Awadi, Jaber Al-Ansari, Ross Maclean, Adrian R. Levy

**Affiliations:** ^1^ICON Epidemiology, 450-688 W Hastings Street, Vancouver, BC, Canada V6B 1P1; ^2^Bristol-Myers Squibb, P.O. Box 454409, Dubai, UAE; ^3^Bristol-Myers Squibb, 5 Research Parkway, Wallingford, CT 06492, USA; ^4^Dubai Hospital, Al Khaleeja Street, Al Baraha, Dubai, UAE; ^5^Precision Health Economics, 11100 Santa Monica Boulevard, Suite 500, Los Angeles, CA 90025, USA; ^6^Department of Community Health and Epidemiology, Dalhousie University, 425-5790 University Avenue, Halifax, NS, Canada B3H 1V7

## Abstract

*Objective*. As little data are available on the quality of type 2 diabetes mellitus (T2DM) care in the Arabian Gulf States, we estimated the proportion of patients receiving recommended monitoring at the Dubai Hospital for T2DM over one year. *Methods*. Charts from 150 adults with T2DM were systematically sampled and quality of care was assessed during one calendar year, using a Healthcare Effectiveness Data and Information Set- (HEDIS-) like assessment. Screening for glycosylated haemoglobin (HbA1c), low-density lipoprotein (LDL), blood pressure, retinopathy, and nephropathy was considered. Patients were classified based on their most recent test in the period, and predictors of receiving quality care were examined. *Results*. Mean age was 58 years (standard deviation (SD): 12.4 years) and 33% were males. Over the year, 98% underwent HbA1c screening (50% had control and 28% displayed poor control); 91% underwent LDL screening (65% had control); 55% had blood pressure control; 30% had retinopathy screening; and 22% received attention for nephropathy. No individual characteristics examined predicted receiving quality care. *Conclusion*. Some guideline monitoring was conducted for most patients; and rates of monitoring for selected measures were comparable to benchmarks from the United States. Greater understanding of factors leading to high adherence would be useful for other areas of preventive care and other jurisdictions.

## 1. Introduction

Due to the rapid increase in the prevalence of type 2 diabetes mellitus (T2DM), particularly in the Middle East and North Africa, it is now recognized as a global public health problem [1]. The prevalence of T2DM in these areas is projected to increase from 9.7% in 2014 to 11.6% by 2035, effectively doubling the current number of cases to close to 70 million when accounting for population growth [1]. In the United Arab Emirates (UAE), the situation is particularly dire [2]; with an estimated 19% to 25% of the population affected, prevalence estimates of T2DM in the UAE are among the highest, globally [1, 3]. Given the large and growing epidemiologic burden, the attendant clinical and economic burden of T2DM management is also steadily increasing [4].

Effective management of T2DM—utilizing a multidisciplinary approach with pharmacotherapy as its foundation—can reduce its associated clinical and economic burden. Decreasing the risk of macro- and microvascular complications due to hyperglycemia is the cornerstone of T2DM therapy [5]. Continuing medical care requires different types of healthcare providers to aggressively manage other cardiometabolic risk factors, including blood pressure and lipid disturbances, in addition to ongoing patient self-management [5]. The clinical benefits of this multidisciplinary approach have been demonstrated in randomized trials of T2DM patients who achieved reduced rates of microvascular complications and other key cardiovascular endpoints, over the long term [6].

For many reasons, T2DM remains inadequately controlled for many of those afflicted. Adherence to medication and ongoing commitment to lifestyle modifications are key determinants of therapeutic success in the management of T2DM [7]; however, poor glycemic control due to clinical inertia of healthcare providers may also play a part in the inadequate control of T2DM. One aspect of the varied efforts required to help improve inadequate control is monitoring of key health indicators and treatment intensification according to consensus recommendations to ensure that quality care is delivered to those afflicted [8]. While data on treatments and health outcomes for persons with T2DM in the Arabian Gulf States are becoming available [9–12], there is still a scarcity of assessments of the quality of care received. The need for robust data to monitor T2DM management in the UAE was recently highlighted in the Lancet [13]; the authors emphasized the challenges facing primary care physicians to improve provision of healthcare, without reliable baseline and comparative data [13]. As it stands, the extent to which the contemporary management of T2DM patients in Dubai meets recommended guidelines is not known.

Tools for measuring the quality of T2DM care have been developed, and their implementation allows for benchmarking within and between countries. The HEDIS (Healthcare Effectiveness Data and Information Set) performance measures developed by the NCQA (National Centre for Quality Assurance) for comprehensive diabetes care are one such set of tools widely used in the United States (US) to measure care quality, predominantly in a commercially insured population [14, 15]. Here, we investigated the level of care being provided to T2DM patients in Dubai, UAE, according to the NCQA HEDIS performance measures, and compared those data to benchmark data on T2DM management from the US. We also sought to determine whether the quality of care received varied according to age and sex, when adjusted for disease duration and severity.

## 2. Material and Methods

This retrospective cohort study was conducted among a sample of persons with T2DM managed at a single diabetes outpatient clinic operating in the Dubai Hospital in Dubai, UAE. This secondary and tertiary care hospital is the largest general medical and surgical hospital in the emirate of Dubai. The study protocol was approved by the Research Ethical Approval Committee at the Dubai Hospital.

### 2.1. Subjects

The target population was persons being treated for T2DM in Dubai, UAE. To be eligible for inclusion, subjects were required to have a diagnosis of T2DM according to American Diabetes Association (ADA) criteria [19], to be 18 years of age or older, and to be of UAE nationality. Subjects enrolled in clinical trials and women who were pregnant during the study period were not eligible.

As part of a larger study of treatment patterns and clinical outcomes [20], electronic medical records of 250 persons with T2DM were systematically sampled from the Dubai Hospital database, by International Classification of Diseases, Ninth Revision (ICD-9) codes (250.x0, 250.x2). That database contains records for the more than 5,000 persons managed at the Dubai Hospital diabetes clinic. Medical charts were screened for eligibility based on subjects attending physician visits at the study site during the study enrolment period, with the enrolment date defined as the subject's most recent visit to the site during that period. Although the study period for the larger study was from October 1, 2009, to September 30, 2011, the NCQA HEDIS assessment is measured over a single calendar year. From the overall sample, the subset of individuals (*HEDIS cohort*) enrolled between October 1 and December 31, 2009, and attending the diabetes clinic at the Dubai Hospital at least once in the 2010 calendar year was identified. As a sensitivity analysis, to allow the use of the data from all 250 patients from the overall sample, the HEDIS assessment was performed using data from a consecutive calendar year following each individual's enrolment date.

The Dubai Hospital is a secondary and tertiary care centre, so subjects may have been initially referred from another clinical site; the study sample would therefore represent a mix of subjects recently diagnosed with T2DM and those with longstanding disease.

### 2.2. Data Collected

In addition to the treatment- and outcomes-specific data reported separately [20], demographic and clinical characteristics from the time of T2DM diagnosis and at study enrolment were collected. The frequency of, and scores on, the measures evaluated in the HEDIS Comprehensive Diabetes Care assessment (glycosylated hemoglobin (HbA1c) screening and control, low-density lipoprotein (LDL) screening and control, blood pressure control, and investigations for retinopathy and nephropathy) were extracted from the charts of eligible individuals. Although core T2DM management (such as assessments of measures of treatment targets) is performed within the Dubai Hospital clinic, some T2DM care (such as renal or retinal) occurs in allied, but not integrated, health clinics.

Data collection was conducted by two trained data abstractors. All case report forms (CRFs) were checked for completeness and those with missing data were checked against the chart. At least 50% of CRFs completed by each abstractor per day were checked against the source data; if discordance between the abstractors was greater than 20%, all CRFs completed that day were validated against their respective chart.

### 2.3. Analysis

Summary statistics were generated for baseline characteristics of the sample. A HEDIS-like assessment, using the NCQA HEDIS Comprehensive Diabetes Care measure, was performed by considering the following on an individual basis over the 2010 calendar year: HbA1c screening, HbAc1 poor control (>9.0%), HbAc1 control (<8.0%), LDL screening, LDL control (<100 mg/dL), blood pressure control (<140/90 mmHg), eye exam performed, and medical attention for nephropathy [7]. The proportion of the HEDIS cohort achieving each of these targets was reviewed against publically available benchmark data from commercial health maintenance organizations (HMOs) in the US.

Individuals were classified as having received comprehensive care in the 2010 calendar year, based on a subset of the HEDIS measures, specifically if HbA1c, LDL, and blood pressure were measured during the period. Using this classification, a multivariable logistic regression model was developed to determine whether the quality of care received varied according to age, sex, or other predictor variables (including weight, relatives with T2DM, need for dialysis, previous kidney treatment, or T2DM duration). Variables significant at *p* < 0.2 in the preliminary multivariable model, which remained significant at *p* < 0.05 in the final model, were retained. Based on the regression model output, odds ratios (ORs) with 95% confidence intervals (CIs) were calculated. As a sensitivity analysis, comprehensive care was considered according to whether HbA1c, LDL, and blood pressure were under control during the study period.

## 3. Results

Of the charts from the 250 subjects included in the larger study, 150 (60%) had a full calendar year of follow-up within 2010 and formed the HEDIS cohort. Approximately one-third of the HEDIS cohort was male, the mean age at enrolment was 58 years, and the mean T2DM duration was 14.2 years. Twenty-five percent of the HEDIS cohort met the ADA [19] and UAE National Diabetes Guideline [16] target level for of HbA1c of <7% at study enrolment (Table 1); and 50% met the NCQA HEDIS target level for HbAc1 of <8% (Table 2). The distribution of the clinical and demographic characteristics of the HEDIS cohort (Table 1) was similar to the distribution of those characteristics in the larger study sample (data not shown).

### 3.1. HEDIS-Like Assessment

The frequency with which members of the HEDIS cohort met each of the NCQA HEDIS assessment targets is presented in Table 2. The frequency of HbA1c and LDL screening among members of the HEDIS cohort in 2010 was high (>90%). While 65.3% of the HEDIS cohort achieved LDL control, the proportion achieving blood pressure (54.7%) and glycemic (50.0%) control was slightly less. Eye exams performed and medical attention for nephropathy were documented in 30.0% and 22.0% of charts of the HEDIS cohort, respectively. Some variability in the frequency of individuals being screened for and achieving targets on individual HEDIS assessment measures was observed by age, but not by sex (Table 3). The sensitivity analysis using the overall sample (*n* = 250) showed that most measures of T2DM quality care did not vary according to whether one calendar year or one consecutive year of data was included per individual, although some differences were seen in frequencies of achieving blood pressure control or receiving medical attention for nephropathy (Table 2).

Seventy-three individuals (48.7%) were classified as having received comprehensive T2DM care, based on having been tested for HbA1c, LDL, and blood pressure during the study period (presented according to age and sex in Table 4). Younger age at enrolment was the only significant predictor of whether an individual received comprehensive T2DM care (OR = 0.966 [95% CI, 0.939–0.994]). Neither age at enrolment nor any of the other potential predictor variables was a significant predictor of whether an individual's HbA1c, LDL, and blood pressure levels were under control during the study period (Table 5).

### 3.2. Comparisons to Benchmarks

The frequency of assessments documented among the HEDIS cohort was reviewed against publically available benchmarks from commercial HMOs in the US (Table 2). The proportion of the HEDIS cohort screened for HbA1c (98.0%) was higher than US benchmarks (89.9%); and while the proportion of the HEDIS cohort achieving glycemic control was slightly less than in the US (50.0% versus 62.3%), the proportion with poor glycemic control was similar between the HEDIS cohort and US benchmarks (approximately 28%). The proportions of the HEDIS cohort undergoing LDL testing (91.3%), and achieving LDL control (65.3%), were also higher than mean US benchmarks (85.6% and 47.7%, resp.). The proportion of those achieving blood pressure control (54.7%), having an eye exam (30.0%), or receiving medical attention for nephropathy (22.0%) in the HEDIS cohort was somewhat lower than mean US benchmarks (65.7%, 57.7%, and 83.6%, resp.).

## 4. Discussion

Although published assessments of clinical outcomes among individuals managed for T2DM in the Arabian Gulf States are increasing, evaluations of the quality of care received remain rare [9–12]. A standardized assessment based on the US HEDIS diabetes care measure was employed to quantify the quality of care received among a sample of T2DM UAE nationals receiving T2DM care from the Dubai Hospital. We found that some guideline monitoring was conducted for all members of the HEDIS cohort. Compared to US benchmarks, rates of HbA1c screening, LDL screening, and LDL control were high; and the proportion of the HEDIS cohort with poor glycemic control was similar to benchmarks. The percentage of participants meeting glycemic targets, receiving medical attention for nephropathy, or receiving retinal examinations was lower among the HEDIS cohort compared to US benchmarks. We found no major or consistent influence of any clinical or demographic factor on the likelihood of receiving quality care or of achieving targets. These data are the first to provide UAE-specific estimates, which can be compared with existing estimates from quality control systems in the US, or offer a starting point for future comparisons of T2DM care with other countries in the Middle East.

Consistent with the findings of the present study, a recent systematic review of T2DM management in the Arabian Gulf States suggested that less than 50% of patients regularly met targets for glycemic control [12]. An evaluation of primary T2DM care from Dubai in 2004 that focused on a slightly different set of clinical measures also identified potential areas for improvement, in terms of patients both achieving targets and also receiving the full set of required investigations [21]. A retrospective study from Abu Dhabi showed that implementing ADA guidelines in a tertiary setting over a three-year period significantly improved T2DM process outcomes; although glycemic control remained suboptimal, improvements in LDL control were seen [10]. The authors postulated that improved LDL control may have partly resulted from updated ADA recommendations for the use of statins in T2DM patients with cardiovascular risk factors or disease [10]; the timing of that recommendation would have also impacted the HEDIS cohort in the present study. Although our study results are not directly comparable due to the varied targets included in the HEDIS measure, our data similarly highlight a role for improved monitoring of T2DM patients in Dubai, both for achieving HbA1c, LDL, and blood pressure targets and also for avoiding microvascular complications.

Findings from the present study demonstrated that younger members of the cohort were more likely than older cohort members to have been screened for HbA1c, LDL, and blood pressure during the study period. Reassuringly, the likelihood of receiving comprehensive diabetes care did not vary by sex; and despite higher rates of screening among younger individuals, it also did not translate to higher rates of achieving guideline targets. There was some variability by age in the proportions of individuals screened or achieving targets on individual test measures, which was not observed when measures were stratified by sex. These were similar to the findings from the study from Abu Dhabi, where more older patients achieved glycemic control and more younger patients reached blood pressure targets [10]. The impact of variability in care on the risk of macrovascular or microvascular complications among those with T2DM in the UAE has not yet been reported. The study also highlights the challenge of achieving more clinically strict benchmarks: 50% of the study patients achieved an HbA1c of <8.0% yet only half those patients lowered their HbA1c to the ADA benchmark of 7.0%. This raises interesting questions as to the optimal means to achieving more aggressive HbA1c control in populations with T2DM.

Preventing microvascular complications is one of the key aims of T2DM management; and given the high prevalence of T2DM in the UAE, early identification of retinopathy and nephropathy could have substantial clinical and economic benefits. The prevalence of diabetic retinopathy was estimated at 19% in a clinical practice-based survey of T2DM patients in Al-Ain [22]; and the prevalence of microalbuminuria, an early sign of diabetic nephropathy, was estimated at 61% in the same sample [23]. Early detection of microalbuminuria, with the use of renal-protective agents, can help reduce the progression of renal disease and cardiovascular events in T2DM [24]; and the risk of both retinopathy and nephropathy increases with increasing age and T2DM duration [22, 24]. The proportion of the HEDIS cohort with examinations reported for retinopathy or nephropathy was low overall, and lower than US benchmarks. Reassuringly, the prevalence of microvascular complications in the HEDIS cohort was also low. At baseline, only 6% of the HEDIS cohort had been diagnosed with retinopathy and 7.3% with nephropathy, less frequent than in the earlier study from Al-Ain [22, 23]. Because renal and retinal care are delivered separately at the Dubai Hospital, low scores on that particular subset of HEDIS measures may indicate an opportunity to improve communication within the healthcare system, in addition to an opportunity to improve the quality of patient care.

This study used real-world data to evaluate the quality of care received by a sample of persons with T2DM managed at a diabetes outpatient clinic in a large hospital in Dubai. Strengths of the study were the implementation of standardized procedures for data collection and analysis; training of data abstractors to ensure consistent methodology; and implementation of quality checks to ensure data completeness, quality, and consistency between reviewers. Although this particular analysis included only a subset of the original sample, the HEDIS cohort well-reflected the overall sample in terms of the demographics and baseline clinical characteristics [20].

There are several potential limitations warranting discussion. First, by including only one urban study site, the care provided here may not be representative of the experiences of all persons with T2DM in the UAE, particularly those from smaller communities. However, the Dubai Hospital is the largest general medical and surgical hospital in Dubai, and this sample would therefore capture a wide cross section of the contemporary T2DM population in the UAE. Conversely, given that the Dubai Hospital is a secondary and tertiary care center, as well as a regional centre for excellence, it is also conceivable that the quality of care may be overestimated compared to primary and peripheral diabetes care centers in Dubai. Outcomes, instead, may be more difficult to achieve among elderly and more complicated diabetic patients. Second, the study population had longstanding T2DM and nearly three-quarters of the subjects were diagnosed more than ten years prior to the study start. Findings from this study may therefore not be generalizable to those with a more recent diagnosis of T2DM with less severe disease and may overestimate the overall burden of and underestimate the quality of care received by persons with T2DM in Dubai. Finally, performing a HEDIS-like assessment requires centralized documentation of all assessments performed, to be able to adequately chronicle all aspects of care received. The study findings may underestimate the actual frequency of assessments for retinopathy and nephropathy among the HEDIS cohort and identify a gap in documentation of care for persons with T2DM in Dubai.

## 5. Conclusions

Benchmarking has been shown to be an effective tool for improving health outcomes among patients with T2DM and is useful for comparisons both within and between countries [25]. Contextualizing contemporary diabetic care in the Middle East through comparison with benchmarks from the US, using assessments such as HEDIS, offers a standardized platform for identifying process and treatment gaps in contemporary T2DM care. A greater understanding of the factors leading to high adherence to guidelines would be useful for other areas of preventive care and other hospitals and jurisdictions. These data may help inform the implementation of interventions to improve clinical outcomes and quality of life for patients with T2DM in this region.

## Figures and Tables

**Table 1:** Demographic and clinical characteristics of T2DM patients in Dubai, followed up for a complete calendar year and therefore eligible for the HEDIS measure, at the time of enrolment in the study, October to December 2009.

Characteristic	T2DM HEDIS patients	
	(*N* = 150)	
	*n*	(%)
Sex		
Male	47	(31.3)
Female	103	(68.7)
Age (years)		
Mean (SD)	58.3	(12.2)
Median (IQR)	56.9	(14.5)
18–34	4	(2.7)
35–49	22	(14.7)
50–64	81	(54.0)
65+	43	(28.7)
Weight^1^ (kg)		
Mean (SD)	81.1	(13.9)
Median (IQR)	81.5	(17.0)
Missing	20	(13.3)
Disease duration (years)^2^		
All patients		
Mean (SD)	14.4	(7.7)
Median (IQR)	14.1	(11.3)
Diagnosed at study site		
Mean (SD)	12.8	(7.6)
Median (IQR)	11.7	(11.2)
<5 years	12	(8.0)
5–9 years	14	(9.3)
10–14 years	14	(9.3)
15–19 years	17	(11.3)
20+ years	9	(6.0)
Diagnosed outside of study site		
Mean (SD)	15.7	(7.5)
Median (IQR)	14.9	(12.4)
<5 years	4	(2.7)
5–9 years	18	(12.0)
10–14 years	20	(13.3)
15–19 years	15	(10.0)
20+ years	27	(18.0)
Meeting HbA1c targets (<7%)^3^		
Yes	38	(25.3)
No	112	(74.7)
Immediate relatives with T2DM		
None/unknown	120	(80.0)
At least one	30	(20.0)
Mother	18	(12.0)
Father	13	(8.7)
Brother	6	(4.0)
Sister	7	(4.7)
Son	0	(0.0)
Daughter	0	(0.0)
Other	2	(1.3)
Prior insulin treatment		
Yes	110	(73.3)
No	40	(26.7)
Number of prior T2DM treatments received^4^		
0	1	(0.7)
1	15	(10.0)
2	46	(30.7)
3	56	(37.3)
4	24	(16.0)
5	8	(5.3)
Type of prior T2DM treatments received^4^		
Metformin	127	(84.7)
Insulin	110	(73.3)
Sulfonylureas	93	(62.0)
Thiazolidinediones	44	(29.3)
DPP-4 inhibitors	11	(7.3)
Meglitinides	4	(2.7)
Alpha-glucosidase inhibitors	2	(1.3)
GLP-1 analogues	1	(0.7)
Metformin + DPP-4 inhibitors	2	(1.3)
Comorbidities/complications		
Any	147	(98.0)
None	3	(2.0)
Macrovascular complications	9	(6.0)
Angina	5	(3.3)
Prior stroke/transient ischemic * *attack	3	(2.0)
Coronary artery disease	0	(0.0)
Peripheral vascular disease	1	(0.7)
Prior myocardial infarction	1	(0.7)
Congestive heart failure	0	(0.0)
Microvascular complications	25	(16.7)
Retinopathy	9	(6.0)
Diabetic nephropathy	11	(7.3)
Chronic kidney disease	9	(6.0)
Diabetic peripheral neuropathy	4	(2.7)
Diabetic foot	1	(0.7)
Chronic renal failure/end-stage * *renal disease	1	(0.7)
Other		
Hyperlipidemia/dyslipidemia	137	(91.3)
Hypertension	119	(79.3)

DPP = dipeptidyl peptidase; GLP = glucagon-like peptide; HbA1c = glycosylated hemoglobin A1c; IQR = interquartile range; kg = kilogram; *n* = number; SD = standard deviation; T2DM = type 2 diabetes mellitus.

^1^Height, abdominal girth, and body mass index (BMI) data are not reported here as they were not routinely recorded in the eligible medical charts. Height was recorded for 20 of the eligible patients; abdominal girth and BMI were only recorded for one patient in the study sample.

^2^Time since diagnosis, calculated as the time from diagnosis date to the study enrolment date.

^3^Treatment target identified by ADA and UAE National Diabetes Guidelines [16, 17].

^4^Unique treatments, received either alone or in combination with other agents.

**Table 2:** HEDIS-like assessment of T2DM care, based upon the HEDIS “comprehensive diabetes care” measure, among T2DM patients in Dubai followed up for a complete calendar year and therefore eligible for the HEDIS measure.

HEDIS CDC measure	Dubai HEDIS cohort		Informal comparison			Sensitivity analysis^1^	
	(*N* = 150)		US commercial HMO HEDIS benchmarks and thresholds^3^ (%)			(*N* = 250)	
	*n*	(%)	Mean	P90	P10	*n*	(%)
HbA1c screening	147	(98.0)	89.9	94.2	85.6	245	(98.0)
HbA1c poor control (>9.0%)^2^	42	(28.0)	27.3	16.8	37.8	65	(26.0)
HbA1c control (<8.0%)	75	(50.0)	62.3	72	52.3	135	(54.0)
LDL screening	137	(91.3)	85.6	91	80	225	(90.0)
LDL control (<100 mg/dL)	98	(65.3)	47.7	57.2	37.2	160	(64.0)
Blood pressure control (<140/90 mmHg)	82	(54.7)	65.7	75.9	52.4	108	(43.2)
Eye exam performed	45	(30.0)	57.7	75.3	40.4	80	(32.0)
Medical attention for nephropathy	33	(22.0)	83.6	89.6	76.9	39	(15.6)

CDC = comprehensive diabetes care; dL = decilitre; HbA1c = glycosylated hemoglobin A1c; HEDIS = Healthcare Effectiveness Data and Information Set; LDL = low-density lipoprotein; mmHg = millimetres of mercury; mg = milligram; *n* = number; T2DM = type 2 diabetes mellitus; US = United States; P90 = 90th percentile; P10 = 10th percentile; HMO = Health maintenance organization.

^1^HEDIS scores are typically calculated for each calendar year. For this HEDIS-like assessment, scores were calculated for the 12 months following the last date of the study accrual period (April 1, 2010, to March 31, 2011).

^2^For this measure, a lower rate signifies better performance.

^3^From the National Committee for Quality Assurance (NCQA) report, State of Healthcare Quality 2011 [18].

**Table 3:** HEDIS-like assessment of T2DM care, based upon the HEDIS “comprehensive diabetes care” measure, among T2DM patients in Dubai followed up for a complete calendar year and therefore eligible for the HEDIS measure, according to age and sex.

	Patients, 18–34 years		Patients, 35–49 years		Patients, 50–64 years		Patients, 65+ years		Patients, males		Patients, females	
	(*N* = 4)		(*N* = 22)		(*N* = 81)		(*N* = 43)		(*N* = 47)		(*N* = 103)	
	*n*	(%)	*n*	(%)	*n*	(%)	*n*	(%)	*n*	(%)	*n*	(%)
HbA1c screening	4	(100.0)	21	(95.5)	79	(97.5)	43	(100.0)	44	(93.6)	103	(100.0)
HbA1c poor control (>9.0%)	0	(0.0)	4	(18.2)	24	(29.6)	14	(32.6)	9	(19.1)	33	(32.0)
HbA1c control (<8.0%)	3	(75.0)	10	(45.5)	42	(51.9)	20	(46.5)	24	(51.1)	51	(49.5)
LDL screening	4	(100.0)	20	(90.9)	73	(90.1)	40	(93.0)	40	(85.1)	97	(94.2)
LDL control (<100 mg/dL)	2	(50.0)	13	(59.1)	58	(71.6)	25	(58.1)	29	(61.7)	69	(67.0)
Blood pressure control (<140/90 mmHg)	4	(100.0)	17	(77.3)	46	(56.8)	15	(34.9)	27	(57.4)	55	(53.4)
Eye exam performed	1	(25.0)	5	(22.7)	26	(32.1)	13	(30.2)	18	(38.3)	27	(26.2)
Medical attention for nephropathy	1	(25.0)	6	(27.3)	14	(17.3)	12	(27.9)	14	(29.8)	19	(18.4)

dL = decilitre; HbA1c = glycosylated hemoglobin A1c; LDL = low-density lipoprotein; mmHg = millimetres of mercury; mg = milligram; *n* = number; T2DM = type 2 diabetes mellitus.

**Table 4:** The frequency of having received comprehensive care, based on a subset of the HEDIS measures, among T2DM patients in Dubai followed up for a complete calendar year and therefore eligible for the HEDIS measure, according to age and sex.

	Comprehensive care (all screening tests performed)							
	Achieved				Not achieved			
	Male (*N* = 21)		Female (*N* = 52)		Male (*N* = 26)		Female (*N* = 51)	
	*n*	(%)	*n*	(%)	*n*	(%)	*n*	(%)
18–34 years	1	(4.8)	3	(5.8)	0	(0.0)	0	(0.0)
35–49 years	5	(23.8)	9	(17.3)	4	(15.4)	4	(7.8)
50–64 years	11	(52.4)	30	(57.7)	12	(46.2)	28	(54.9)
65+ years	4	(19.0)	10	(19.2)	10	(38.5)	19	(37.3)

**Table 5:** Univariate logistic regression coefficients for a model estimating the association between age, sex, and other explanatory variables on whether an individual received comprehensive care (all screening tests performed), among T2DM patients in Dubai followed up for a complete calendar year and therefore eligible for the HEDIS measure.

Variable	Received comprehensive care							
	HbA1c, LDL, and blood pressure measured				HbA1c, LDL, and blood pressure controlled			
	Coefficient	SE	*z* value	*p* value	Coefficient	SE	*z* value	*p* value
Male sex	0.233	0.353	0.659	0.510	−0.551	0.427	−1.290	0.197
Weight (kg)	0.006	0.012	0.484	0.628	−0.003	0.156	−0.172	0.863
Number of relatives with T2DM	−0.573	0.415	−1.379	0.168	−0.762	0.468	−1.630	0.103
Need for dialysis	−14.630	882.740	−0.017	0.987	−147.030	1455.400	−0.012	0.991
Previous kidney transplant	−0.761	1.236	−0.616	0.538	−0.754	1.243	−0.607	0.544
Age at enrolment	−0.035	0.015	−2.365	0.018	−0.021	0.017	−1.212	0.226
Time since diagnosis	0.009	0.021	0.407	0.684	−0.015	0.028	−0.544	0.586

HbA1c = glycosylated hemoglobin; LDL = Low-density lipoprotein; SE = standard error; kg = kilograms; T2DM = type 2 diabetes mellitus.
